# Defining a prevalence level to describe the elimination of Lymphatic Filariasis (LF) transmission and designing monitoring & evaluating (M&E) programmes post the cessation of mass drug administration (MDA)

**DOI:** 10.1371/journal.pntd.0008644

**Published:** 2020-10-12

**Authors:** Benjamin S. Collyer, Michael A. Irvine, T. Deidre Hollingsworth, Mark Bradley, Roy M. Anderson

**Affiliations:** 1 Department of Infectious Disease Epidemiology, School of Public Health, Faculty of Medicine, St Mary’s Campus, Imperial College London, London, United Kingdom; 2 Institute of Applied Mathematics, University of British Columbia, Vancouver, Canada; 3 Big Data Institute, Li Ka Shing Centre for Health Information and Discovery, Nuffield Department of Medicine, University of Oxford, Oxford, United Kingdom; 4 Global Health Program, GlaxoSmithKline (GSK), Brentford, United Kingdom; University of Zurich, SWITZERLAND

## Abstract

The global decline in prevalence of lymphatic filariasis has been one of the major successes of the WHO’s NTD programme. The recommended strategy of intensive, community-wide mass drug administration, aims to break localised transmission by either reducing the prevalence of microfilaria positive infections to below 1%, or antigen positive infections to below 2%. After the threshold is reached, and mass drug administration is stopped, geographically defined evaluation units must pass Transmission Assessment Surveys to demonstrate that transmission has been interrupted. In this study, we use an empirically parameterised stochastic transmission model to investigate the appropriateness of 1% microfilaria-positive prevalence as a stopping threshold, and statistically evaluate how well various monitoring prevalence-thresholds predict elimination or disease resurgence in the future by calculating their predictive value. Our results support the 1% filaremia prevalence target as appropriate stopping criteria. However, because at low prevalence-levels random events dominate the transmission dynamics, we find single prevalence measurements have poor predictive power for predicting resurgence, which suggests alternative criteria for restarting MDA may be beneficial.

## Author summary

The disease lymphatic filariasis is caused by parasitic worms that are spread by mosquito vectors, and is endemic across much of Sub-Saharan Africa, Asia, South and Central America, with over 893 million people in 49 countries considered to be at-risk. In 1997 the Global Programme to Eliminate Lymphatic Filariasis was established by the WHO with the aim of eliminating the disease as a public health problem by 2020 and supplies recommended guidelines for national programs to implement. The recommended control strategy is to annually administer deworming drugs to the population at-risk until stopping criteria based on disease prevalence are met, after which the disease prevalence is periodically measured to determine if transmission is ongoing. In our study, we simulate the disease transmission in a synthetic population, both during a mass drug administration program and after it is stopped, to statistically evaluate the effectiveness of the current guidelines. We focus on the ability of prevalence measurement post-MDA cessation to predict bounce-back or elimination by estimating the predictive value of antigenemia and filaremia surveys.

## Introduction

Lymphatic Filariasis (LF) is one of the most important neglected tropical diseases that persists in resource poor settings throughout Sub-Saharan Africa, Asia, South and Central America. An estimated 1.3 billion people are at risk for contracting LF, and prior to intensive control efforts in many countries, 120 million people were infected [1]. LF is a disease that occurs when infective larvae of the nematode parasite are transmitted to a human host via mosquito vector feeding. The insect vector is an intermediate host within which parasite development takes place before transmission to the definitive human host. Three types of filarial nematode worms cause LF; namely, *Wuchereria bancrofti*, *Brugia malayi*, and *Brugia timori*. Of these, *W*. *bancrofti* is the most prevalent filarial infection worldwide being responsible for about 90% of reported LF cases [2].

The World Health Organization (WHO) considers LF as a candidate for elimination due to advances in diagnosis and treatment, and the availability of drug donations from the pharmaceutical industry [1]. These advances paved the way for the formulation in 1993 of a global elimination strategy. In 1997, the World Health Assembly passed Resolution 50.29, calling for the elimination of LF as a public health problem. In 2000 the World Health Organization (WHO) established the Global Programme to Eliminate Lymphatic Filariasis (GPELF) with the goal of eliminating the disease as a public health problem by 2020 [3, 4]. The programme has two key goals: namely; first to use community-wide annual mass drug administration (MDA) to interrupt transmission, using a combination of albendazole and ivermectin in areas co-endemic with onchocerciasis, and albendazole and diethylcarbamazine (DEC) elsewhere; and second to alleviate suffering by managing morbidity and preventing disability in clinical LF patients.

These goals are defined in the WHO’s 2020 Neglected Tropical Disease (NTD) Road Map and the London Declaration on NTDs [5, 6]. They are currently undergoing revision in the process of defining a new road map for 2020 to 2030.

LF control has been one of the major successes of the WHO NTD programme with a number of countries declaring elimination following intensive community-based MDA programmes. Since 2000, a cumulative total of 7.1 billion treatments have been delivered to more than 890 million people at least once. To-date sixteen countries (Cambodia, The Cook Islands, Egypt, Kiribati, Maldives, Marshall Islands, Niue, Palau, Sri Lanka, Thailand, Togo, Tonga, Vanuatu, Viet Nam and Wallis and Fortuna and Yemen) are now validated as having achieved elimination of lymphatic filariasis as a public health problem. Seven additional countries have successfully implemented recommended strategies, stopped large-scale treatment and are under surveillance to demonstrate that elimination has been achieved. Preventive chemotherapy is still required in 49 countries but has not been delivered to all endemic areas as of the end of 2017. However, in 2019 WHO estimates that 886 million people in 49 countries worldwide remain threatened by lymphatic filariasis and require preventive treatment. Strategic objectives were established by WHO for interrupting transmission by 2020 but these targets have not been achieved in many countries. The objectives address the specific challenges of initiating MDA (and sustaining high coverage), other interventions, or both, in all endemic areas, scaling up these interventions to full geographical coverage, stopping interventions when transmission has been interrupted, establishing effective surveillance after MDA has stopped, and verifying success.

Elimination of LF as a public health problem as defined by WHO, is reducing infection prevalence in an area to below a target threshold and providing the recommended basic package of care in all areas with lymphoedema or hydrocele patients. A process of validation is used for formal confirmation of elimination as a public health problem. As of the end of 2019, WHO acknowledged that the evidence documented in dossiers received from 16 countries met the validation criteria. The goal of community wide MDA is to reduce Mf prevalence within a sub-area to below 1% (or alternatively prevalence measured by antigen-based tests, which have lower specificity, to below 2%), after which a period of monitoring and evaluation (M&E) commences. During this period, transmission assessment surveys (TAS) are conducted every 2–3 years to assess if bounce-back of transmission is occurring [7].

In November 2018, the WHO published data showing that a total of 465.4 million people received treatment for lymphatic filariasis during 2017, in 37 countries that implemented the WHO-recommended large-scale community wide treatment (mass drug administration; MDA) of populations at risk of the disease [8]. WHO guidelines recommend 4–6 annual rounds of MDA, consisting of a combination of albendazole and either Ivermectin or diethylcarbamazine, with a minimum coverage level of 65%.

In 2018, WHO published a new guideline on alternative MDA regimens and recommended a 3-drug regimen (known as IDA) to accelerate the elimination of lymphatic filariasis as a public health problem [9]. Since its release, 4 countries, including Samoa, have initiated country wide implementation of IDA; an additional 6 countries introduced IDA in 2019 [10]. Expansion of elimination programmes and eventual uptake of IDA by eligible countries mean that millions of people should no longer require treatment for this debilitating neglected tropical disease by 2030.

A key issue in assessing any future strategy for LF control concerns what is the threshold prevalence that should be chosen as indicating a cessation in transmission within a defined country or setting. The current choice of 1% Mf positive prevalence (filaremia), or alternatively 2% circulating filarial antigen positive prevalence (antigenemia), has not been explored in a formal statistical sense, to see if when that prevalence is achieved what fraction of those communities that achieve the target will indeed move to transmission elimination as opposed to ‘bounce back’. In a stochastic world–there will be a probability that below these thresholds, transmission elimination has occurred in the absence of the immigration of infectives (people and vectors). In addition, past work on the transmission dynamics of LF suggests that this threshold may depend on a variety of local conditions such as the value of the basic reproductive number, R_0_, within any given location. R_0_ is a direct measure of the pristine transmission intensity prevailing prior to the introduction of MDA programmes. Past work on other human helminth infections suggests that the critical prevalence level will also depend on what assumptions are made concerning key variables such as adult worm life expectancy in the human host and adult worm aggregation within the human host population (as measured inversely by the negative binomial *k* value) [11–13].

In this paper we return to the issue of what is a sensible prevalence threshold at which to declare that the interruption of transmission has taken place, looking at parameter sensitivity and employing a framework that defines a probability that a given prevalence level will result in the cessation of transmission. We also examine how best to monitor what is happening post cessation of MDA in terms of the frequency and the interval between monitoring and evaluation surveys. Two recent papers have reviewed the evidence for LF transmission elimination. The first, by Davis and colleagues, examines the dependence of the chance of elimination on certain model parameters, using a simple model [14]. The second, a recent paper by Prada and colleagues modelling transmission of LF post MDA-cessation, has calculated the sensitivity and specificity of various monitoring thresholds [15]. We build on this analysis by considering three different baseline transmission settings, and by considering the predictive value of different monitoring thresholds, a statistic that accounts for the context in which the prevalence threshold is used [14]. We employ an individual based stochastic model developed by Irvine and co-authors [12] and use the approaches to define transmission threshold prevalence levels that have been employed for other human helminth infections such as the schistosome parasites and the soil transmitted helminths [16–18].We address four research questions relating to the use of community wide MDA to stop the transmission of the nematode parasites that cause LF. These are as follows:

1. Once prevalence is lowered below 1% filaraemia (the current WHO guideline) by repeated rounds of MDA, how likely is it that elimination of transmission (which we define as no subsequent new infections) occurs, and conversely how likely is it that ‘bounce back’ occurs?
2. What level of prevalence during the monitoring and evaluation period lets us best predict the likelihood of both transmission elimination and bounce-back?
3. How does the length of time between MDA cessation and starting M&E affect the ability to predict elimination and bounce-back?
4. How sensitive is the prevalence threshold to parameter assignments, especially those that are difficult to measure or estimate from epidemiological data?

## Methods

### Ethics statement

This study uses previously published data. All data used are anonymized and unidentifiable. IRB approval was not requested.

### Parameter estimation

In this study we use TRANSFIL (the name of the programme developed by *Irvine et al*), an individual based stochastic LF transmission model, to simulate LF endemics during and post MDA [12]. The model calculates changes over time and patient age in the number of male and female worms, and microfilaria (Mf) carried within each individual human host, as well as the size of the L3 larval population in the mosquito vectors. Human population-level heterogeneity, to generate a negative binomial probability distribution of adult worms per human host, is generated by a randomly distributed exposure risk factor assigned at birth (resulting in the compounding of a series of Poisson distributions where the Poisson means follow a gamma distribution). It is also assumed that exposure to mosquito bites is age dependent, and that sexual reproduction of worms within the human host is completely polygamous.

We have used baseline data on the filaremia prevalence of infection, stratified by age obtained from three communities, Ngahmbule and Yauatong in Papua New Guinea (see Table 1 in [19]), and Malindi in Kenya (see Table 2 in [20]) to estimate rates of age dependent exposure to infection in the human host These three datasets were chosen because they represent three different levels of baseline endemicity. Ngahmbule and Yauatong, representing moderate and high baseline endemicity, are located in the Dreikikir region of Papua New Guinea, where data was collected in 1994 prior to a five year MDA intervention. The data from Malindi, Kenya was conducted at the beginning of a two round MDA program conducted 2002–2004. Using this data we fit the vector-to-host ratio parameter (*V*:*H*), which controls the intensity of the endemic (directly proportional to the magnitude of the basic reproduction number R_0_); the parameter *k*, which measures inversely the aggregation of parasites in the human host population; the parameter a_max_ which determines the shape of the age intensity profile; and *r*, which determines the rate of the density dependent saturation of microfilaria in the mosquito population.

Our fitting routine uses an approximate Bayesian computation methodology (commonly referred to as ABC), which is a likelihood-free and flexible parameter inference method [21] (see additional materials for details).

### Other model parameters

Table 1 contains the model parameters used and the source references of the estimates.

**Table 1:** Model parameters.

Symbol	Parameter	Value	Source
n	Size of population	1000	
λ	Number of bites per mosquito	10 per month	[19, 22]
V:H	Ratio of vectors to hosts	Fitted	
a_max_	Maximum exposure age	Fitted	
ψ_1_	Proportion of L3 leaving mosquito per bite	0.414	[23]
ψ_2_	Proportion of L3 leaving mosquito that enters host	0.32	[24]
s_2_	Proportion of L3 entering host that develop into adult worms	0.00275	[25, 26]
μ	Death rate of adult worms	0.0104 per month (7 years life expectancy–in sensitivity analyses a 5–9 year range is explored)	[27]
α	Production rate of Mf per worm	0.2 per month	[28]
γ	Death rate of Mf	0.1 per month	[29]
g	Proportion of mosquitos which pick up infection when biting an infected host	0.37	[30]
σ	Death rate of mosquitos	5 per month	[24]
k	Aggregation parameter of individual exposure to mosquitos	Fitted	
r	Parameter controlling the saturation of Mf that enters a mosquito	Fitted	
κ	Maximum density of Mf that enters a mosquito after biting.	4.395	[31]
h(a; a_max_)	Rate at which individuals of age a are bitten	Linear from 0 to a_max_ with maximum of 1	[25]
χ_1_	Proportion of Mf killed for an individual MDA round using ALB and DEC	0.95	[32, 33]
χ_2_	Proportion of adult worms sterilised using ALB and DEC	0.55	[32, 34]
ρ	Systematic adherence of MDA (0 –no systematic adherence, 1- fully systematic)	0.25	
ρ_c_	Coverage of MDA	0.65	
τ	Death rate of human population	0.00167 per month	
Δt	Time step	1 month	

### Model description

The transmission model utilised in TRANSFIL is a stochastic individual host based model which builds on the pioneering work of EPIFIL, the age-structured deterministic LF transmission model which consists of a set of partial differential equations with derivatives for time and human host age, and LYMFASIM, an alternative stochastic individual based model [35, 36]. The event table for the individual based stochastic model in TRANSFIL is given in Table 2.

**Table 2:** TRANSFIL model event definitions with the stochastic simulation framework. Where necessary, the subscript *i* indexes individual human hosts in the population.

*Event*	*Description*	*Frequency*
Worm acquisition from mosquito bites (per host)	*new_males*_*i*_, *new_females*_*i*_ ~ Poisson (0.5 *λ V*:*H ψ*_*1*_ *ψ*_*2*_ s_2_ h(a_*i*_) *L3 b*_*i*_ *Δt*) *male_worms*_*i*_ *→ male_worms*_*i*_ *+ new_males*_*i*_ *female_worms*_*i*_ *→ female_worms*_*i*_ *+ new_females*_*i*_	Each month
Worm death (per host)	*male_deaths*_*i*_ ~ Poisson (*μ male_worms*_*i*_ *Δt*) *female_deaths*_*i*_ ~ Poisson (*μ female_deaths*_*i*_ *Δt*) *male_worms*_*i*_ *→ male_worms*_*i*_*−male_deaths*_*i*_ *female_worms*_*i*_ *→ female_worms*_*i*_*—female_deaths*_*i*_	Each month
Mf production (per host)	*Mf*_*i*_ *→ Mf*_*i*_ *+ α Δt female_worms*_*i*_ I*(male_worms*_*i*_ *> 0)*	Each month
Mf deaths (per host)	*Mf*_*i*_ *→ Mf*_*i*_*−γ Δt Mf*_*i*_	Each month
Host deaths	u_*i*_ ~ Uniform(0,1) Host dies if (1 –exp(-τ Δt)) < u_*i*_	Each month
Host births	Equal to host death	Each month
Host risk factor assignment	*b*_*i*_ ~ Gamma (*k*,*1/k*)	At host birth
Mean uptake of larvae	$\bar{L}=\sum_{i}L({Mf}_{i})b_{i}/\sum_{i}b_{i}$ (where *i* indexes hosts) *L*(*m*) = *κ*(1−*exp*(−*r m/κ*))^2^	Each month
L3 in reservoir (per mosquito)	L3 = λ g $\bar{L}$ / (σ + λ ψ_1_)	Each month
MDA	*male_worms*_*i*_ *→ male_worms*_*i*_ *(1- χ*_*1*_*)* *female_worms*_*i*_ *→ female_worms*_*i*_ *(1- χ*_*1*_*)*	Every 12 months
Mf prevalence measurement (per host)	$I(Poisson\;({Mf}_{i})>0)$	As required
Antigen prevalence measurement (per host)	$I(({male\_worms}_{i}+{female\_worms}_{i})>0$	As required

### Prevalence thresholds

It is instructive to distinguish between three different possible prevalence thresholds:

Stopping threshold–a prevalence threshold which is used to determine whether MDA can stop or if further rounds are required. The WHO recommended stopping threshold is 1% by filaremia or 2% by antigenemia. We note that the 1% filaremia target is supported by evidence from previous modelling studies, whereas the 2% antigenemia target has been introduced as a conservative estimate of the equivalent level of antigenemia [7].
Monitoring threshold—a prevalence threshold that is used while in the monitoring period to decide whether MDA needs to restart. The TAS monitoring threshold is 2% antigenemia in 6–7 year olds.
Critical threshold–a prevalence level which is a feature of deterministic transmission models, below which the disease cannot sustain itself and elimination is certain. In models of helminth transmission, the existence of a critical threshold is created by the required presence of both sexes of adult worm within host for reproduction (this is also known as a reproductive Allee effect). The modelled critical threshold can be used to guide the choice of stopping and monitoring thresholds, however, it should be used with caution, because at low prevalence individual-level variation and infrequent chance biting events are expected to drive transmission, and these cannot be accounted for by the deterministic models.

### Diagnostics

WHO guidelines recommend that during MDA programs, LF prevalence should be monitored at sentinel and spot-check sites to determine if the level of infection is below the stopping threshold. Prevalence is determined either by using thick blood smears from which microfilaria counts (per volume of blood) are recorded, or diagnostics that detect the circulating filarial antigen (Ag) produced by the *W*. *Bancrofti* parasite.

The current recommended antigen-based diagnostic is the Alere Filariasis Test Strip (FTS). Measuring filaremia from blood smears is both expensive and labour intensive because the periodicity of *W*. *Bancrofti* requires samples being taken at night, between 10 pm and 2 am. The inconvenient sampling time can lead to the issues of low participation and non-compliance, which may ultimately reduce the reliability of the data collected. Measuring antigenemia rapidly at point-of-care is both cheaper and logistically simpler to perform than measuring microfilaremia using blood smears.

Mf counts conducted by well-trained practitioners are considered to have 100% specificity and have high sensitivity for moderate and high intensity infections. The sensitivity is presumed to be greatly reduced for low intensity infections. The FTS diagnostic has high specificity and specificity (~90%), even for low-intensity infections [37]. However, there is some evidence *W*. *Bancrofti* antigens can remain in the body for 2–3 years after the infection has been cleared, which may reduce the specificity of the test immediately following an intervention [38].

After MDA has been halted, the prevalence of LF is monitored by the TAS, where the recommended diagnostic is the FTS only, and the sample population is formed from 6–7 year olds. 6–7 year olds are chosen because it is assumed that antigen detected in young children will indicate new infections, and is unlikely to be pre-existing antigen produced by infections which occurred pre-MDA cessation. Although Mf-counts are not recommended by the WHO for the TAS, we have also produced results where TAS utilise mf-counts, as a comparator to the antigen based diagnostic.

### Design of the stochastic individual based simulations

In each location setting, MDA is implemented annually for the number of years required to reduce the mean prevalence across the set of simulations, as measured by Mf counts in thick blood smears to below the 1% Mf stopping threshold. Under this design not all realisations of the stochastic process, which represents transmission on a village scale, will reach the 1% filaremia stopping threshold before MDA cessation. This design is reflective of the real situation where the decision to stop MDA is based on sentinel sites and not measurements of prevalence in every location. Community wide treatment is performed with a 65% coverage level spread uniformly by age group, and a moderate level of systematic non-compliance (the correlation of non-compliance to treatment between rounds was chosen to be 0.25 to represent a moderate degree of non-compliance). We model compliance by assigning each individual a random number *u*_*i*_, drawn from a Gaussian distribution with mean u_0_ and variance *σ*^*2*^, chosen to fix the overall coverage, *ρ*_*c*_, and correlation between rounds, *ρ* (for details see [12]). Individuals are treated on each round if independent draws from a standard Gaussian with mean *u*_*i*_ are less than zero.

We record the prevalence as measured by filaremia (sampled from 50% of the population) and antigenemia (sampled from 6–7 year olds) 6 months after MDA ceases, and subsequently annually for 8 years post MDA cessation. For a given prevalence level monitoring-threshold, we then calculate the positive (PPV) and negative predictive values (NPV), for predicting the future elimination of LF transmission 40 years post MDA cessation [17, 18].

The positive predictive value for a given monitoring threshold is equal to the probability that transmission is eliminated 40 years post MDA cessation, given that the measured prevalence is below the threshold. The negative predictive value for a given monirotinge threshold is equal to the probability that transmission persists 40 years post MDA cessation, given that the measured prevalence is above the threshold. A low PPV will result in wasted resources, as the effort to reduce the prevalence to below the stopping threshold might need to be repeated. A low NPV could result in over-treatment in communities which would have reached elimination with no further rounds of MDA. Ideally, the chosen monitoring thresholds should have reasonably high values (>0.9) for both the PPV and NPV statistics, to give program managers confidence in decisions taken based on the results of prevalence measurements.

## Results

### Parameter estimates and summary of simulations

Table 3 summarises the parameters found by the model fitting routine for the three locations. Posterior distributions for each of the fits may be found in the S1 Supporting Information. The maximum a posteriori (MAP) estimates of the parameters were used to generate our results.

**Table 3:** MAP estimates of parameters, with 95% credible intervals in brackets, and estimated R_0_ (R_0_ is defined as the average number of female parasites produced by a single female parasite over the course of an average lifespan, that themselves survive to have offspring, in the absence of density dependent effects).

	k	V:H	r	a_max_	R_0_
Malindi, Kenya	0.15 (0.11–0.26)	60.0 (45.4–74.9)	0.14 (0.09–0.24)	32.0 (17.4–36.6)	2.12
Ngahmbule, PNG	0.48 (0.38–0.81)	101.0 (98.6–112.1)	0.24 (0.18–0.49)	20.3 (18.2–22.7)	5.10
Yauatong, PNG	1.66 (1.29–2.11)	167.1 (161.8–197.9)	0.12 (0.09–0.17)	1.7 (0.8–2.7)	8.20

A total of 10,000 realisations were performed for each location. For Malindi Kenya, 7 rounds of MDA were required to reduce the mean Mf prevalence to 1% from a baseline of 20%, with 82% reaching transmission elimination within 40 years. Eight (8) rounds of MDA were required for the mean Mf prevalence in the Ngahmbule location to go below 1% filaremia, starting from a baseline of 50%, with 88% of runs reaching elimination within 40 years. In the Yauatong location nine (9) rounds of MDA were required for the mean Mf prevalence to go below 1%, starting from a baseline of 88% filaremia, with 99.5% of runs reaching elimination within 40 years.

**Fig 1:**
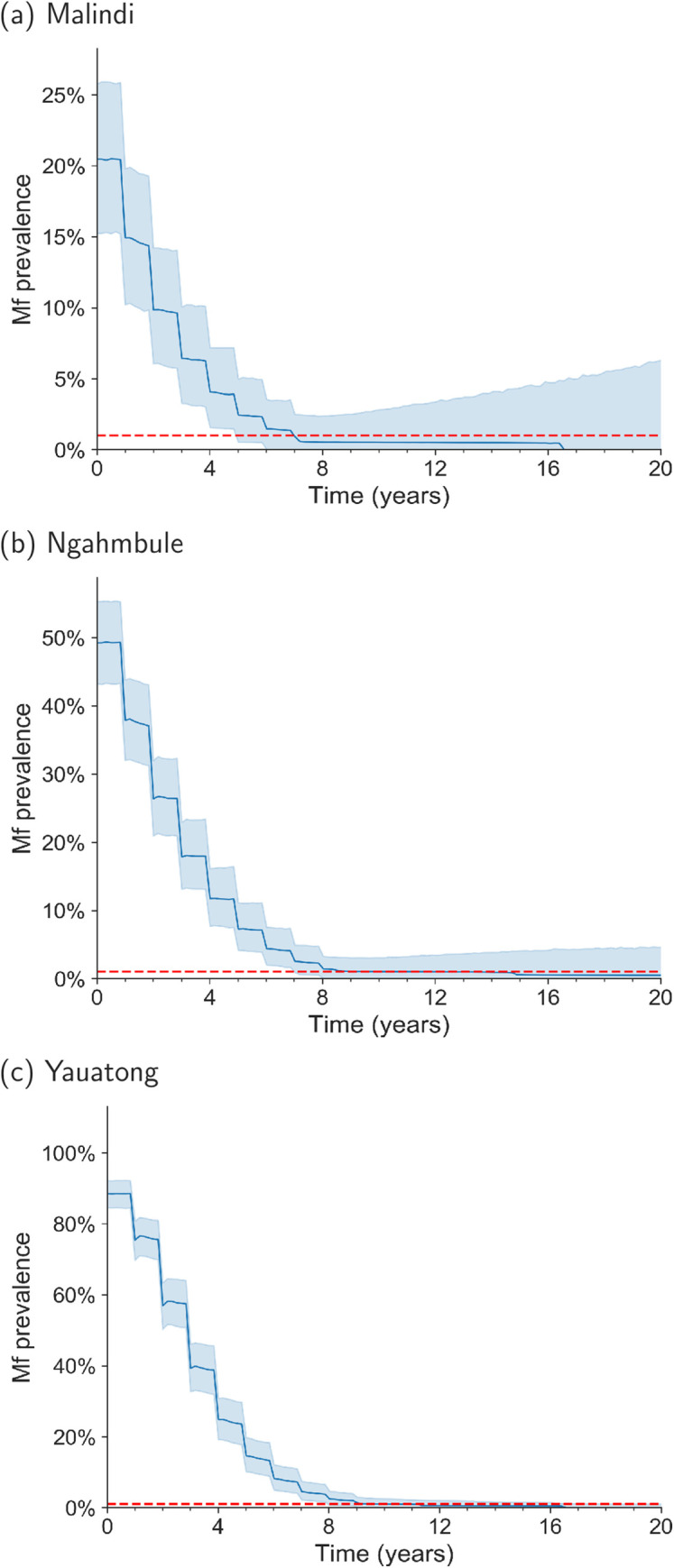
Mf prevalence time-series for (a) Malindi (Kenya) with 7 annual MDA rounds (b) Ngahmbule (PNG) with 8 annual MDA rounds and (c) Yauatong (PNG) with 9 annual MDA rounds. The first round of MDA occurs at year 1 in each setting. The blue lines are the median of the distribution, the shaded regions represent 95% of the distribution. Red dashed line represents 1% Mf stopping threshold.

Fig 1 shows the decline in prevalence, measured by filaremiea through successive rounds of MDA in each parameter setting. A larger proportion of realisations bounce-back in the Malindi setting than the Papua New Guinea settings. The apparent relationship between higher baseline prevalence and an increased chance of elimination after reaching the stopping targets through MDA, appears to run contrary to the deterministic mathematical theory that a higher R_0_ should decrease the value of the unstable equilibrium (the transmission breakpoint), however this theory is only applicable if other model parameters are held constant [39]. The observed pattern can be understood by considering the effect of the aggregation of Mf adult worms in the host population after successive MDA rounds on the theoretical breakpoints. Previous modelling studies have demonstrated that higher adult worm aggregation decreases the breakpoint in deterministic models, and conversely lower adult worm aggregation results in a higher breakpoint [31]. This effect is equivalent to the phenomenon of ‘super-spreaders’ in microparasite disease, the presence of whom can make the control of an epidemic more challenging [40]. At equilibrium, adult worm aggregation is closely related to the mosquito-exposure aggregation parameter, *k*, however the aggregation of adult worms increases as successive rounds MDA are administered (this is a consequence of imperfect coverage and non-compliance, which leaves a proportion of the population untreated). Therefore, we can expect the coverage, compliance and number of rounds of MDA to influence the chance of elimination, as well as R_0_ and the mosquito-exposure aggregation parameter, *k*.

**Fig 2:**
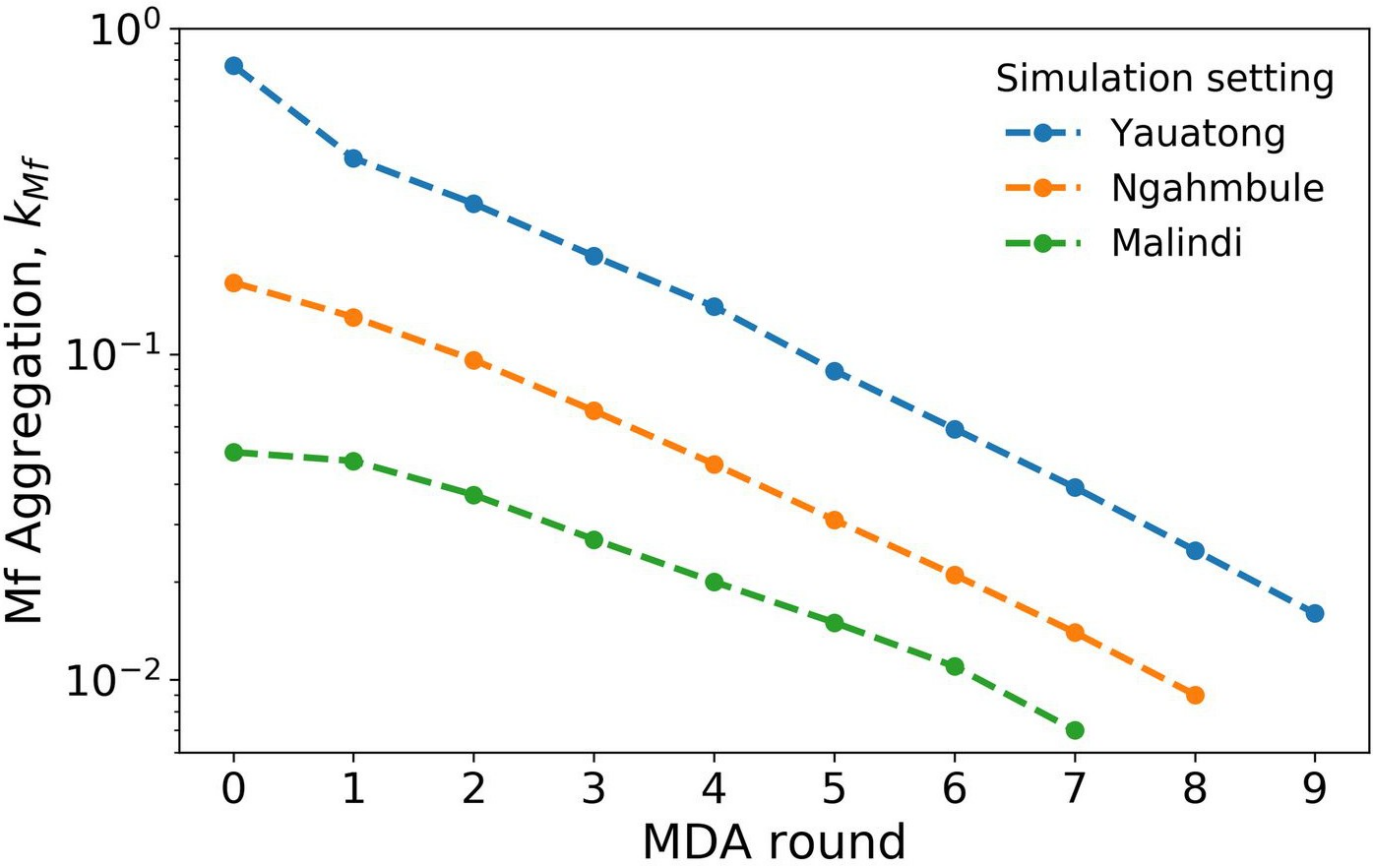
Aggregation of simulated Mf counts after each round of MDA, estimated using maximum likelihood estimation (MLE). Markers represent the mean MLE from 1000 realisations. Round 0 corresponds to the aggregation estimated at baseline.

Fig 2 shows maximum likelihood estimates of the aggregation of simulated Mf counts in blood smears, after each round of MDA, for each simulation setting. The microfilaria counts (which may be used as a proxy for adult worms) are far less aggregated for the Yauatong parameter setting than for the other settings by the final MDA round (at which time the mean Mf prevalence is just below 1% in each setting), which explains why a greater percentage of the runs reach elimination. These results suggest that the effect of higher mosquito-exposure aggregation outweighs effect of lower R_0_ on the chance of elimination post MDA cessation, and that the additional rounds of MDA required in high R_0_ settings are not enough to mitigate this.

### Predictive values at different thresholds

**Fig 3:**
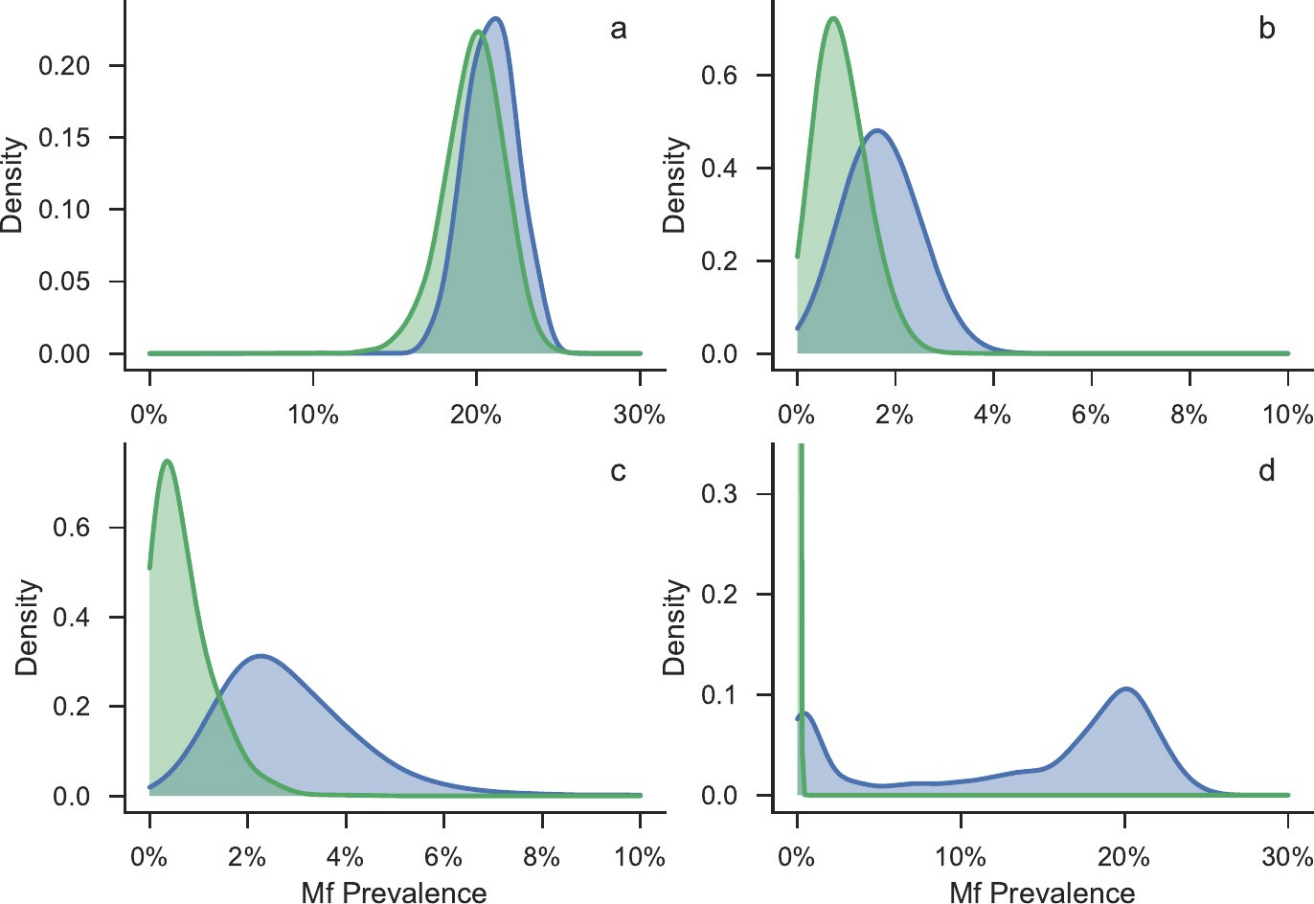
Snapshots (made using kernel density estimation) of the Mf prevalence distributions (green- realisations that eliminate, blue–realisations that bounce-back), at different times for fit to Malindi, Kenya data a) at baseline (pre-intervention) b) 6 months post intervention c) 5 years post intervention d) 40 years post MDA cessation.

**Fig 4:**
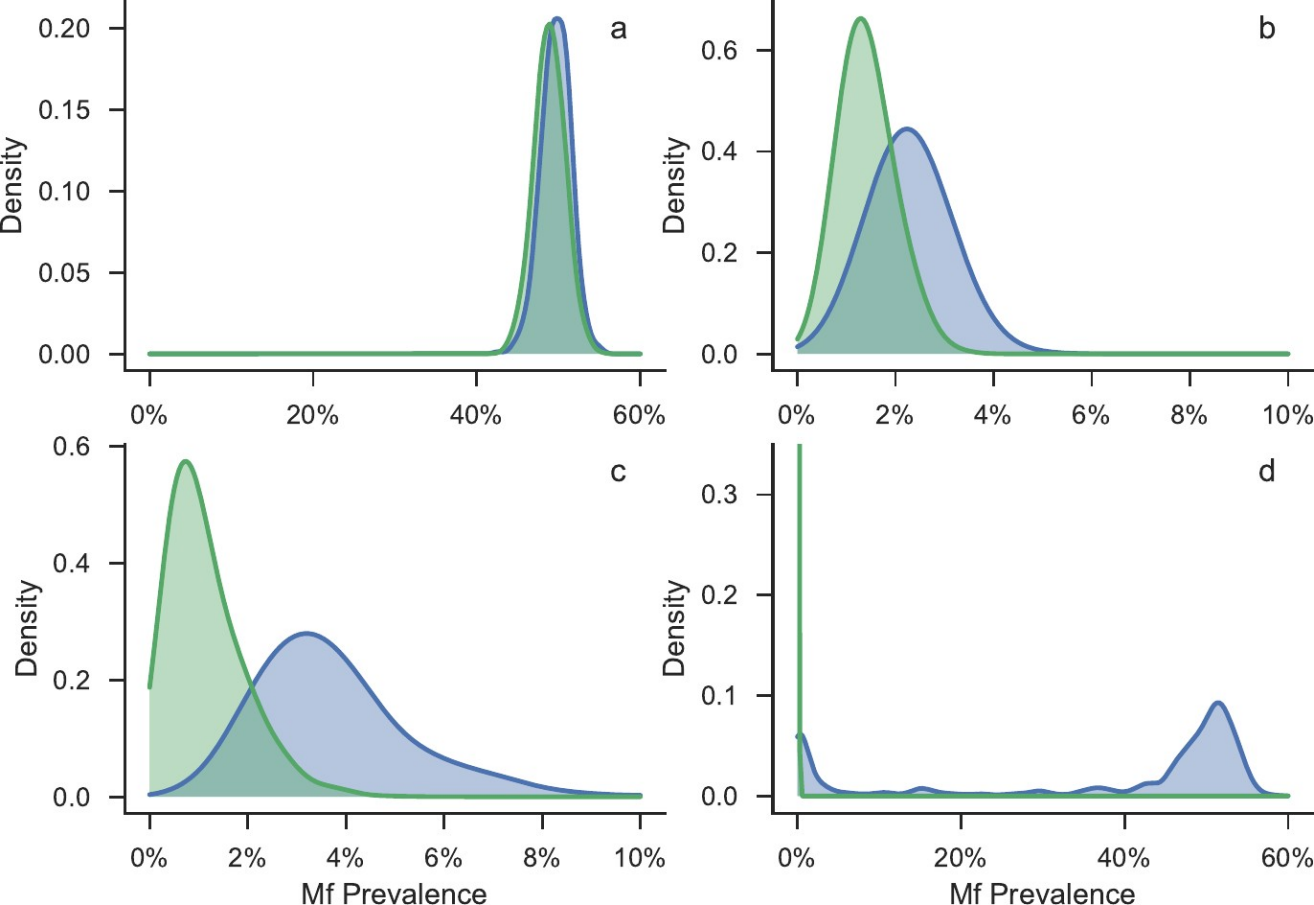
Snapshots (made using kernel density estimation) of the Mf prevalence distributions (green- realisations that eliminate, blue–realisations that bounce-back), at different times for fit to Ngahmbule, PNG data a) at baseline (pre-intervention) b) 6 months post intervention c) 5 years post intervention d) 40 years post MDA cessation.

Fig 3 and Fig 4 show snapshots of the filaremia distributions of realisations that are eliminated after 40 years in green, and those that are not eliminated in blue, at (a) at baseline (b) six months post MDA cessation (c) 5 years post MDA cessation, and (d) 40 years post MDA cessation. In both figures panel (a) shows that at baseline the distributions almost entirely overlap, hence it is impossible to accurately predict which realisations are likely to be either eliminated or bounce back purely from measurements of prevalence at baseline. As time passes, the distributions separate, allowing for the possibility of predicting the final outcome from the prevalence alone.

**Fig 5:**
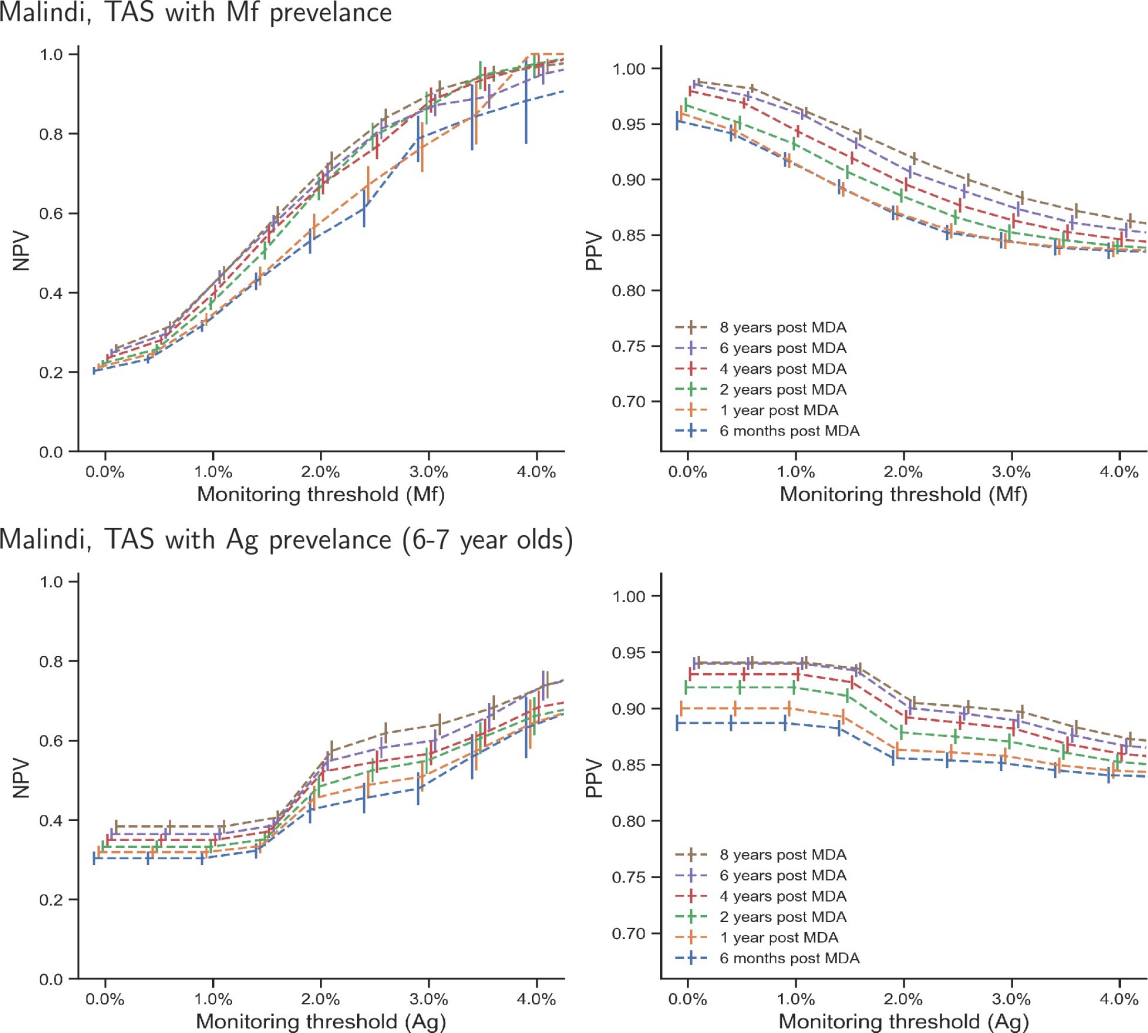
Negative predictive values (for predicting bounce-back, given the measurement is above the threshold) and positive predictive values (for predicting elimination 40 years post MDA cessation, given the measurement is below the threshold), at different antigenemia (6–7 year olds) and filaremia monitoring thresholds, and at different measurement times (shown in varying colours). Parameter setting—Malindi, Kenya. Bars show 95% confidence intervals. To aid visualization, the bars have been offset in the x-axis -0.1% to 0.1% in steps of 0.04%.

**Fig 6:**
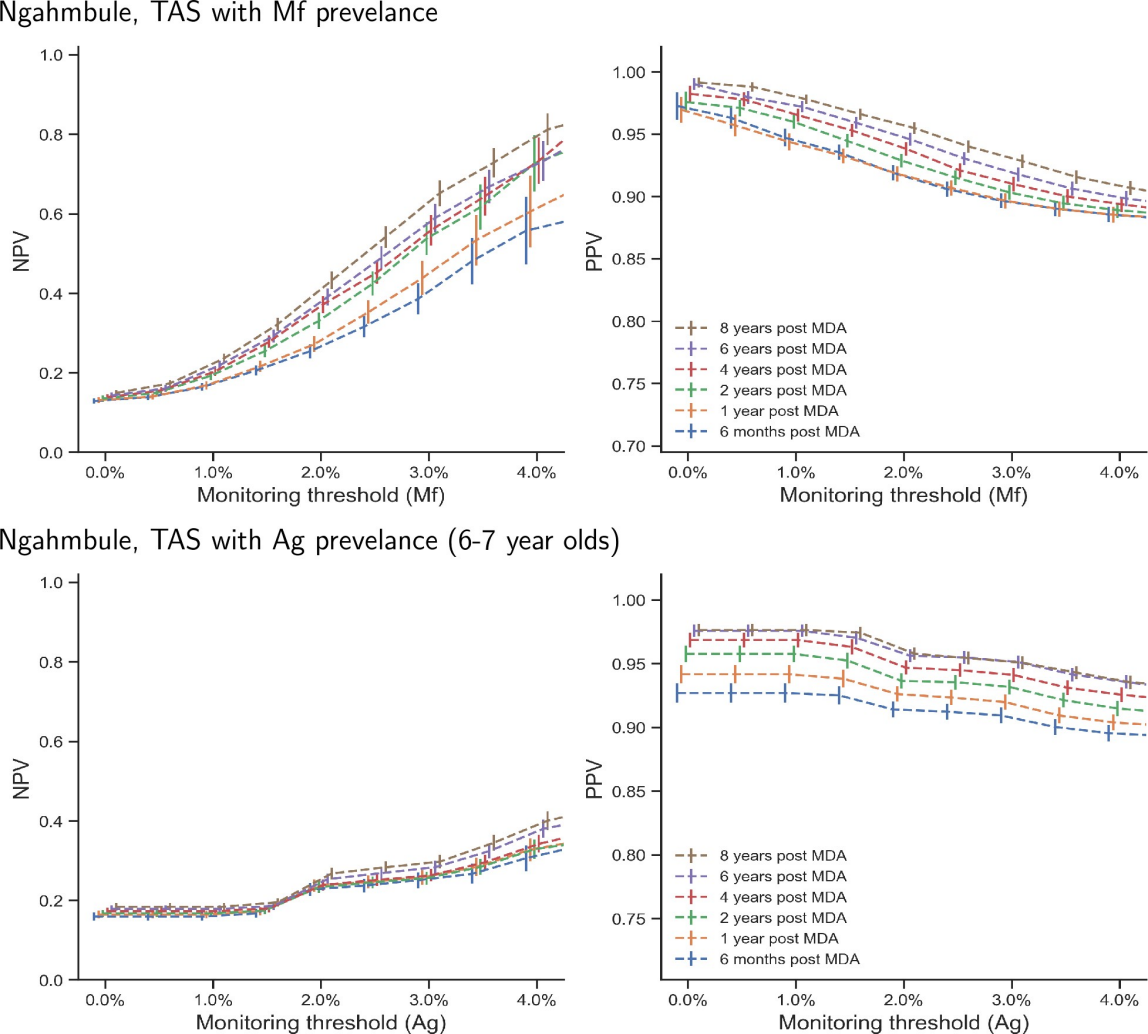
Negative predictive values (for predicting bounce-back, given the measurement is above the threshold) and positive predictive values (for predicting elimination 40 years post MDA cessation, given the measurement is below the threshold), at different antigenemia (6–7 year olds) and filaremia monitoring thresholds, and at different measurement times (shown in varying colours). Parameter setting—Ngahmbule, PNG. Bars show 95% confidence intervals calculated using estimates of the standard error, which depends on the number of realisations above or below the threshold. To aid visualization, the bars have been offset in the x-axis -0.1% to 0.1% in steps of 0.04%.

Fig 5 and Fig 6 show the positive and negative predictive values at varying monitoring thresholds for Malindi and Ngahmbule respectively, at different time points, using antigenemia testing (left) and filaremia testing (right). In both locations, predicting elimination is generally easier than predicting bounce-back, as indicated by PPVs that are generally larger than the NPVs. This is because the number of realisations that reach elimination is much larger than those which do not, which means there are fewer false negatives (measured prevalence below the monitoring threshold with subsequent bounce back) than false positives.

The observed relationship between predictive values and monitoring thresholds also differs between the two diagnostic methods; the relationship when the diagnostic uses Mf counts has a smooth sigmoidal shape, conversely when the antigenic diagnostic is used, the relationship appears to have steps. This is likely to be a consequence of adult worms featuring in the model as discrete entities, whereas the microfilaria are modelled as a continuous concentration within the blood.

We find the PPVs for the Mf-count diagnostic are similar to those where the TAS is conducted using antigen diagnostics for 6–7 year olds, when using the monitoring threshold is above 1.5% for each diagnostic. Below a 1.5% monitoring threshold, filaremia in the population provides more positive predictive value than antigenemia in 6–7 year olds. In the Malindi setting (Fig 4), the 1% filaremia monitoring-threshold provides a PPV of above 0.9 at all sampling times, whereas PPVs above 0.9 are only achieved for antigen diagnostics when the sample is taken over two years post MDA cessation, and the antigenemia threshold is below 1.5%. If the antigenemia monitoring-threshold is 2%, then PPVs are only found to be greater than 0.9 if the sample is taken more than two years post MDA cessation. In the Ngahmbule parameter setting (Fig 5), 1% filaremia provides a PPV above 0.9, and both 2% and 1% antigenemia also provide a PPV above 0.9.

In general, increasing the time one waits after MDA cessation to measure prevalence improves the PPV at each threshold prevalence. This extra predictive value gained while waiting comes at the cost of larger increases in prevalence for those than are not eliminated. However, at the 1% Mf threshold, there is only approximately a 5% improvement in PPV between measurements at 6 months and 8 years post treatment cessation, indicating that the increased predictive power is unlikely to be worth further future rounds of MDA if the prediction is incorrect.

Conversely, the 1% fileremia and 2% antigenemia monitoring-thresholds result in poor NPV (predictive value for predicting bounce-back). The Malindi parameter setting gives NPVs of less than 0.5 and 0.6 for 1% fileremia and 2% antigenemia thresholds, respectively, whilst the Ngahmbule location has NPVs less than 0.25 for these thresholds. This means that despite having a prevalence value higher than the 1% and 2% monitoring thresholds, even 8 years post-intervention, most realisations will decay to elimination within 40 years without further intervention. This indicates that a higher threshold prevalence, or decision criteria that use more than one measurement, could be beneficial when deciding whether or not to restart rounds of MDA.

### Sensitivity to parameter assignment—mean adult worm lifespan

**Fig 7:**
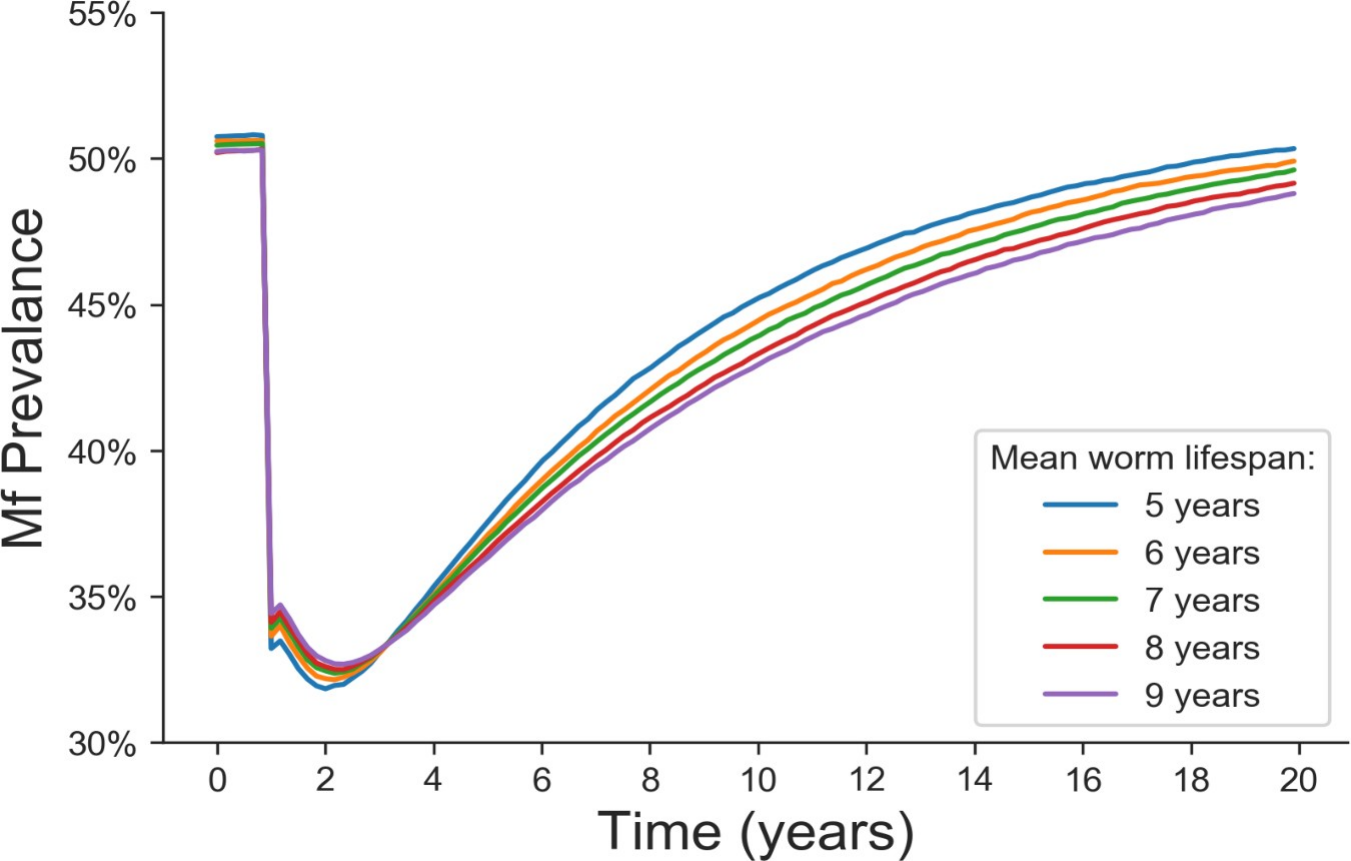
Time-series of Mf prevalence after a single round of MDA, illustrating how the mean lifespan of adult worms affects the speed of bounce-back.

Simulation studies based on the individual based stochastic model LF transmission predict that both the speed and probability of elimination after reducing the prevalence to below 1% by repeated annual rounds of MDA will depend strongly on the mean adult worm lifespan (see SI and Fig 7). This is important, because the estimates for this parameter differ greatly within the literature. It is prohibitively difficult to measure in-vivo worm lifespans directly; hence only indirect evidence exists. This evidence has been obtained either from the decay rate of prevalence in areas post interruption of transmission, or from the duration of Mf production of individual patients who have left endemic regions [41–44]. These different methods have resulted in substantial variance in published estimates. The variation in estimates may be due to the inadequacies of the estimation methodologies. However, it is also plausible that large variation may exist spatially between different host reservoirs, as the lifespan of the adult worms may depend on many factors in the complex two host life cycle of the parasite and the environment in which transmission is taking place. Therefore, it is prudent to determine what effect varying this parameter has on our results.

An increased mean worm lifespan decreases the time gradients of the dynamics, which will have two effects in the stochastic system. The first is that the average decay times to either the stable endemic equilibrium or elimination are longer, the second is that fluctuations will play a more important role in the long-term dynamics of an individual realisation. The interaction of these two effects is non-trivial, so we have investigated the effect of varying the lifespan in the range 5–9 years on the PPV and NPVs, with the V:H ratio parameter adjusted to control for changes in the basic reproduction number R_0_.

**Fig 8:**
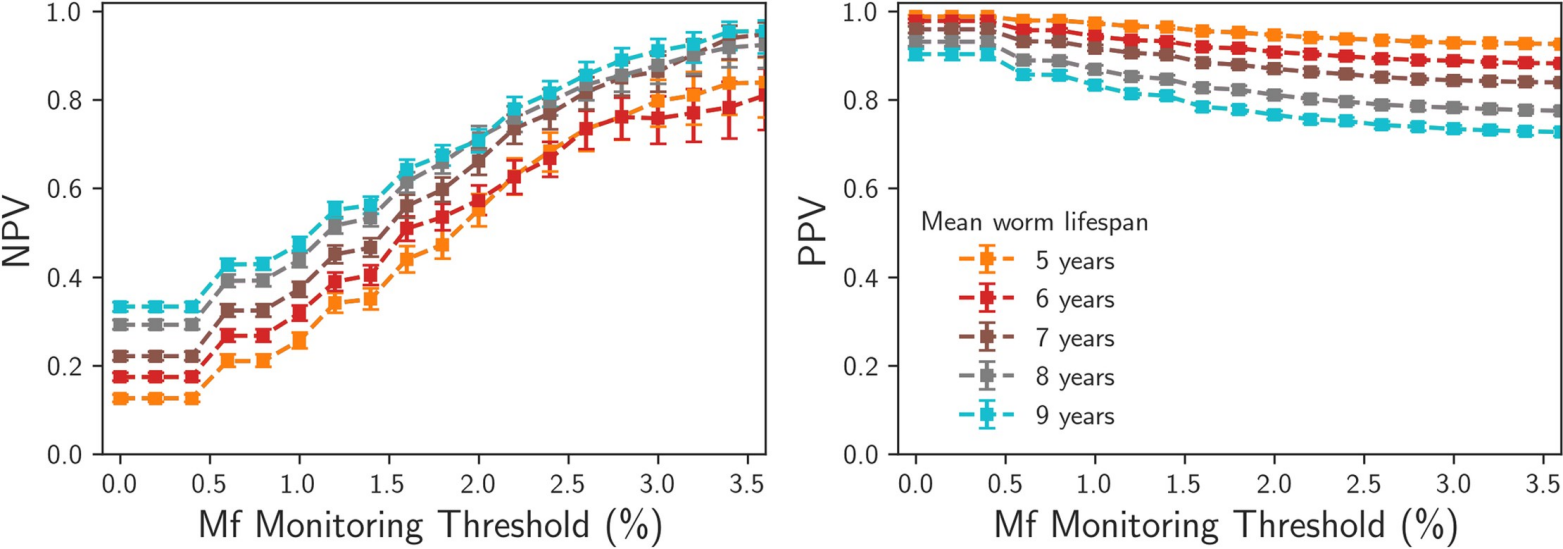
Predictive values (left: NPVs for predicting bounce-back, right: PPVs for predicting elimation), for TAS prevalence measurement performed 2 years post MDA cessation, Malindi setting, with mean worm lifespans ranging from 5 years (dashed line) - 9 years (solid line).

**Fig 9:**
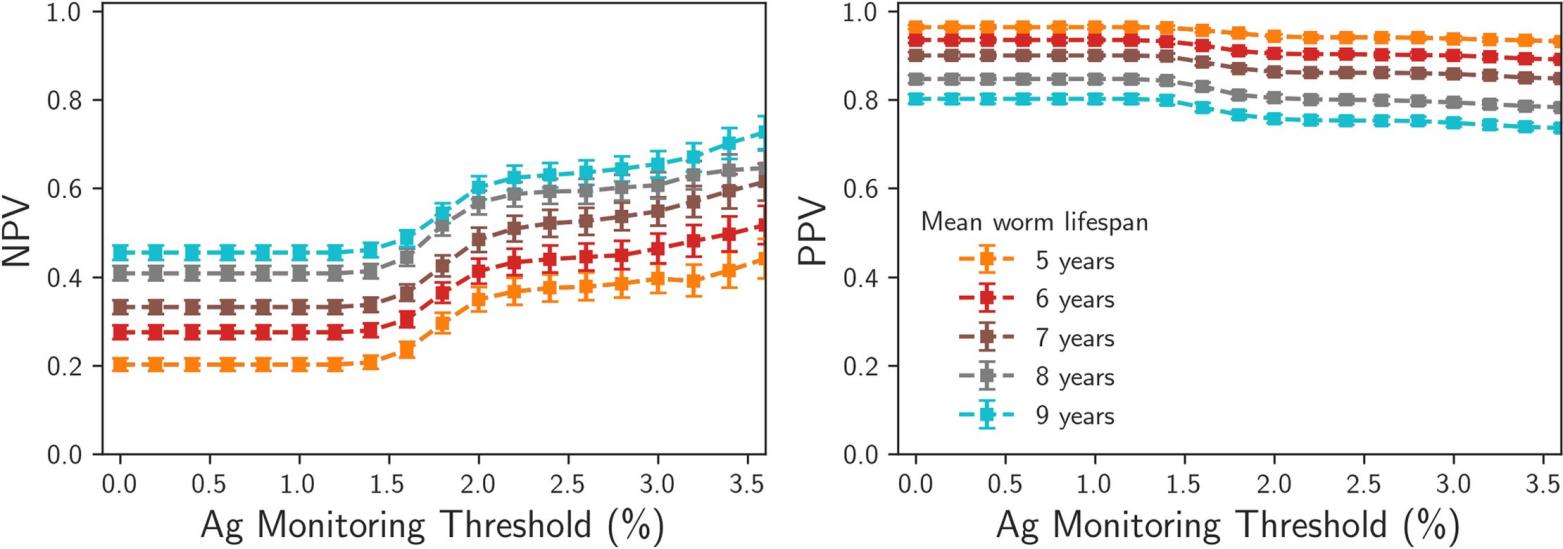
Predictive values (left: NPVs for predicting bounce-back, right: PPVs for predicting elimination), for TAS prevalence measurement performed 2 years post MDA cessation, Ngamhbule setting, with mean worm lifespans ranging from 5 years (dashed line) - 9 years (solid line).

In Figs 8 and 9 we see that increasing the mean lifespan has a negative effect on the PPVs, making the long-term prediction of elimination from positive evaluations at given Mf prevalence thresholds more difficult. The PPV values at the 1% Mf prevalence threshold are greater than 0.8 for both the Malindi and Ngahmbule locations, and at 2% Ag prevalence thresholds are greater than 0.7. We find that uncertainty from the worm lifespan parameter alone is able to reduce the confidence in predictions of elimination from prevalence measurements.

Conversely, we find that the effect of increased worm lifespan is to increase the NPVs. This is counter-intuitive, as one might expect that the increased lifespans should make long-term behaviour less predictable. The NPVs increase with increased lifespan simply because a greater number of realisations are able to persist after 40 years post MDA cessation.

## Discussion

In this paper we have used TRANSFIL, a stochastic individual-based model, with parameters fitted to data from three epidemiological settings, to examine the likelihood of bounce-back and elimination post MDA cessation. The model mechanistically captures key facets of the transmission dynamics, including sexual reproduction, the density dependent uptake of microfilaria by the mosquito vectors, and heterogeneous burdens of infection among the human population.

Our results indicate that the aggregation of infections is highly influential in determining the chances of eradication. Consequently and counter-intuitively, the chance of elimination may be higher in a some high transmission settings with limited aggregation than in low transmission ones. This is because infections are necessarily aggregated in lower-transmission settings, since lower transmission and an evenly spread risk will result in an a more unstable endemic (in this scenario the infected individuals would have burdens with few mating pairs, and chance events could able to stop the production of microfilaria more easily). Consequently lower-transmission settings are much more dependent on a few higher-risk individuals for ongoing transmission, and these individuals are more likely to have been missed by MDA or be re-infected, than if risk was spread more homogeneously, as is possible in high transmission settings. The implication of this is that while good monitoring and evaluation practices post-MDA is essential everywhere, they are especially vital for areas where the biting risk is highly aggregated. The three settings we modelled suggest that lower stable baseline prevalence could be an indicator for high aggregation, but this requires further investigation.

We find that once MDA has stopped after reaching the 1% Mf stopping-threshold, if filaremia is subsequently measured and found to be lower than 1%, or antigenemia in 6–7 year olds less than 2%, then elimination is highly likely to occur within 40 years without further intervention (PPVs > 0.9 at 1% filaremia monitoring-threshold, PPVs >0.85 at 2% antigenemia moniroting-threshold). This suggests that 1% filaremia is a suitable prevalence threshold to determine the cessation of MDA programs, and gives support to the current GPELF strategy. However, some assessment of the measurement error in Mf diagnostics to measure prevalence are required especially at low average intensities of infection. If this widely used diagnostic underestimates prevalence after repeated rounds of MDA have reduced the average intensity if infection, a somewhat lower value for the Mf count test might be appropriate.

Both filaremia and antigenemia above 1%/2% monitoring-thresholds, however, are not reliable predictors of bounce-back. This is because at low levels of prevalence, close to the theoretical deterministic breakpoint in transmission, we find that stochastic noise dominates the dynamics, and so increases or decreases in the prevalence are not necessarily indicative of long-term trends. NPVs of around 0.9 are achieved in the Malindi (Kenya) setting with monitoring-thresholds > 3% Mf prevalence, however are not achieved using antigenemia in the range of thresholds examined (0% - 4%). NPVs greater than 0.9 are not achieved in the Ngahmbule setting using both filameria or antigenemia monitoring thresholds in the ranges considered. This suggests that restarting additional rounds of MDA may only be necessary if a measurement of Mf prevalence is found to be greater than prevalence thresholds which are in excess of 4%. However, we note that the geographical unit for TAS surveys are areas termed evaluation units (often referred to as EUs), and can be formed from several smaller ‘implementation units’ (IUs) which are usually at the district or village scale. This means that surveys conducted at the EU scale which return results above or below certain thresholds may hide districts where the real prevalence is significantly below or above the threshold respectively. For this reason, a sensible degree of caution would need to be exercised if using a higher threshold to determine restarting MDA than the threshold used to determine when to stop. This is especially the case if the diagnostic underestimates the prevalence.

The lack of predictive power at low monitoring thresholds for predicting bounce-back is consistent with the findings of Prada and colleagues, who found that single prevalence levels have poor specificity [15]. They propose a TAS methodology that detects the difference in prevalence between surveys, and show that it is able to provide greater specificity. Decision criteria that are able to use data from more than one survey will inevitably have greater predictive power, however, care should be taken not to create success/failure criteria that produce counter-intuitive decisions. For example, if TAS failure is determined purely by an increase in prevalence between surveys, then an area may be required to restart MDA with lower prevalence than other areas which have experienced only modest decline in prevalence from a higher initial measurement. This may lead to a perceived inequity of treatment. Failure criteria that rely on the absolute prevalence of two surveys may help avoid this possible issue.

Our results show that the PPVs and NPVs increase with the duration between MDA cessation and the prevalence measurement, although the increases are generally smaller after 2 years post cessation. This suggests that in order to identify areas that are at risk of bounce-back, it is sufficient to conduct TAS surveys 2 years post MDA cessation, with low risk of large increases in the prevalence in that time. To accurately determine the optimal spacing between tests, an economic cost-effectiveness analysis study would be necessary to weigh-up the costs of performing each evaluation, against the benefit of the early identification of bounce-back trajectories.

There are several areas where data is limited and model behaviour/structure is sensitive to differing assumptions on parameter values:

- We have not included human migration in the model. Immigration into and out of the study area is likely to have an influence on transmission, particularly as elimination is being approached. The net consequence of immigration could either be the effective addition of a source of infective material that acts as an additional external reservoir, reducing the chance of elimination, or an effect that acts to dilute the prevalence within the area, which may increase the chance of elimination. Which effect, and the magnitude of that effect, is dependent on human sociological and demographic factors at play in the area of interest.
- Our model does not include an acquired immunity effect in the transmission dynamics, the inclusion of which could act to increase the strength of transmission after prevalence in the population has been supressed for several years. The nature of acquired immunity to filarial infection and how it develops as individuals are repeatedly exposed as they age, is not well understood, and additional research in this area is required before models are adapted to incorporate some form of acquired immunity [45].
- Age and mosquito-exposure are the only sources of heterogeneity included in TRANSFIL. It may be reasonable to expect that over-dispersion of the L3 larval stage burden in the mosquito population also plays a significant role in the dynamics at low prevalence. The addition of empirical data to parameterise within-mosquito burden will improve accuracy of the model.
- Our results only consider the control of LF transmission via preventative chemotherapy, and do not consider additional benefits that may be expected if chemotherapy is combined with other control methods, e.g. insecticide treated bed-nets or DEC-fortified salt. Increased use of alternative control strategies, or any change in human behaviour which reduces biting risk, will act to reduce the effective reproductive number R (which is proportional to the Vector/Human host ratio) and hence hasten the decline in prevalence and decrease the chance of bounce-back.
- The blood smear diagnostic simulated in this study, where capillary blood is taken at night and examined under microscope, assumes a Poisson distributed count of microfilariae [30]. It is known that concentrations of microfilariae within the blood are highly sensitive to the time of day, and so it is plausible that this diagnostic will have greater overdispersion than the Poisson distribution. More data availability in this area where variability is measured between samples taken at the same time, and between different days, will improve the accuracy of the model.
- We can expect that the sample size used to estimate prevalence will influence the predictive values; larger sample sizes should increase both PPVs and NPVs.

Finally, this paper has not considered monitoring practices post-TAS. A well designed TAS methodology should minimise the risk of resurgence after being completed, however, there can be no absolute guarantees of what will occur in the medium to long term post completion. Policy makers should consider how best to continue monitoring in such settings, and well calibrated models may help design appropriate methodology.

## Supporting information

S1 Supporting Information ABC parameter fits. (DOCX)
