# Supplementary material for: Defining a prevalence level to describe the elimination of Lymphatic Filariasis (LF) transmission and designing monitoring & evaluating (M&E) programmes post the cessation of mass drug administration (MDA)
Source: PLoS Negl Trop Dis. 2020 Oct 12;14(10):e0008644. doi: 10.1371/journal.pntd.0008644 (PMC7549789; doi:10.1371/journal.pntd.0008644)
Supplement: S1 Supporting Information — (DOCX) [file pntd.0008644.s001.docx]

**Supporting Information S1: ABC parameter fits**

An approximate Bayesian computation (ABC) parameter estimation method was used to fit the aggregation parameter, *k*, the vector to host ratio, *V:H*, the parameter *a_max_* and parameter,*r*, to data from Malindi (Kenya), Ngahmbule and Yauatong (Papua New Guinea). The data comprises age binned prevalence at baseline, and overall prevalence after rounds of MDA (with given coverage levels). We used an unweighted L2 distance function to measure the distance between simulated data and real data, and used an adaptive rejection algorithm with decreasing tolerances. The data for Ngahmbule and Yauatong can be found in Table 1 of [1]. The data for Malindi can be found in Table 2 of reference [2]. The python source code for producing the fits can be found at https://github.com/sempwn/ABCPRC . Figure S1, S3 and S5 show the posterior distributions of the fits in each of the setting. Figure S2, S4, and S6 show the validation of the fits with 100 runs using maximum a posteriori (MAP) estimator parameters against the real data.


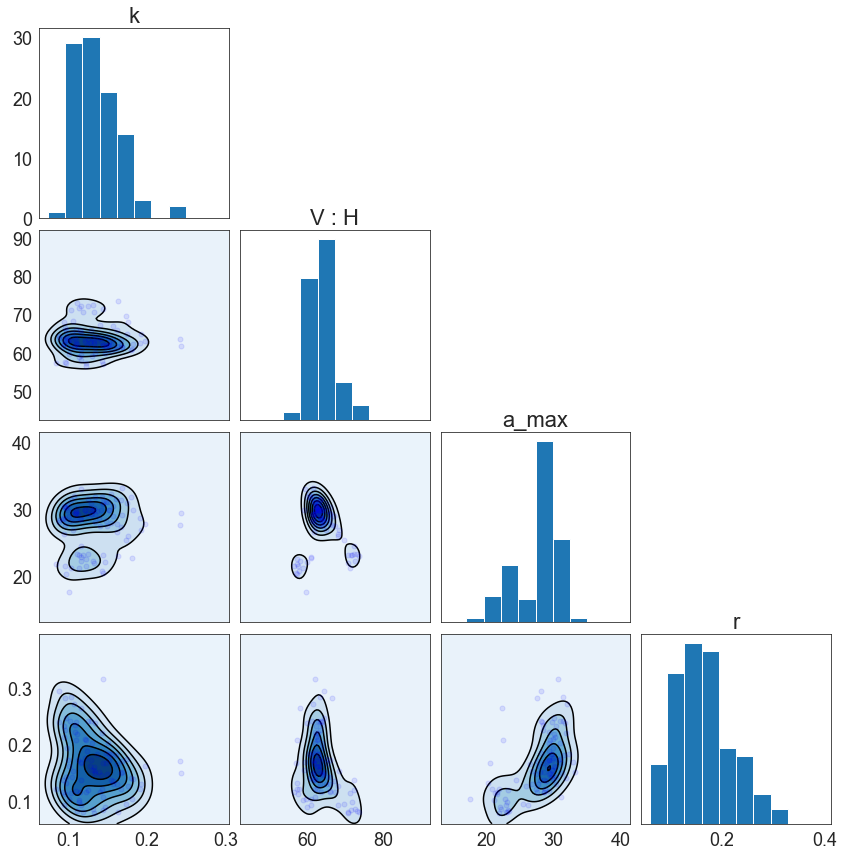


**Figure S1:** **ABC parameter fits for Malindi (Kenya) dataset.**

**
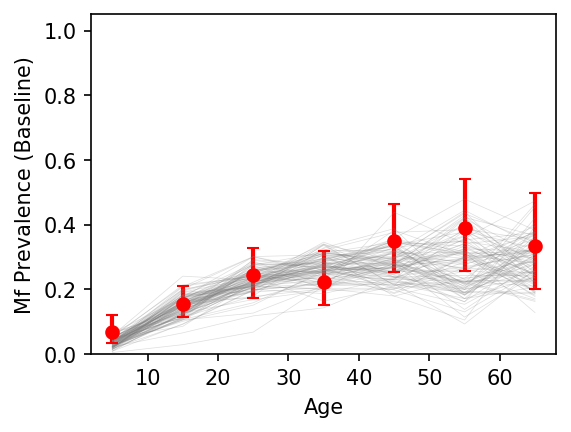

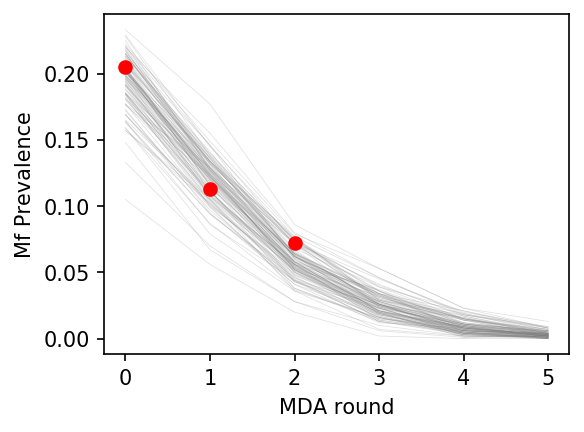
**

**Figure S2:** **100 realisations of TRANSFIL, with MAP parameters (*k* = 0.15, *V:H = 60.0*, *a_max_* = 32.0 , *r = 0.14*) and Malindi baseline data (red).**

**
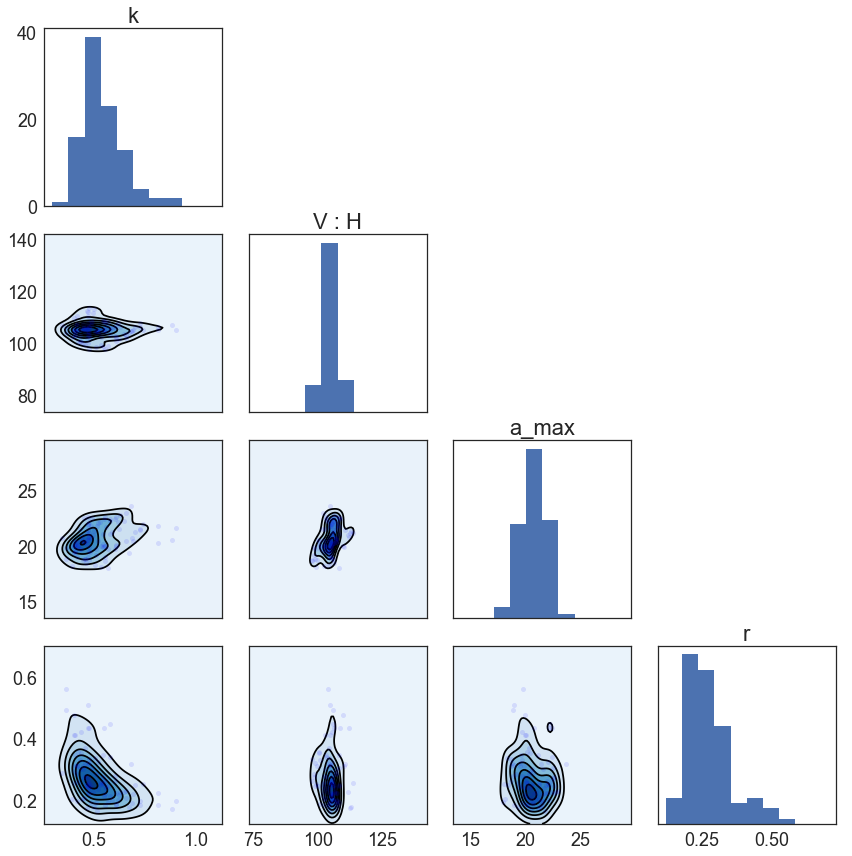
**

**Figure S3: ABC parameter fits for Ngahmbule (PNG) dataset.**

**
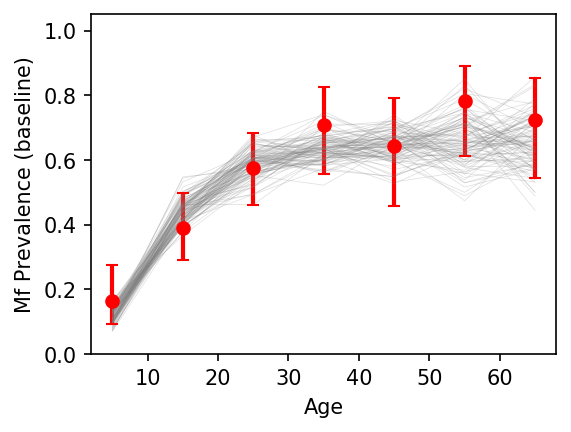

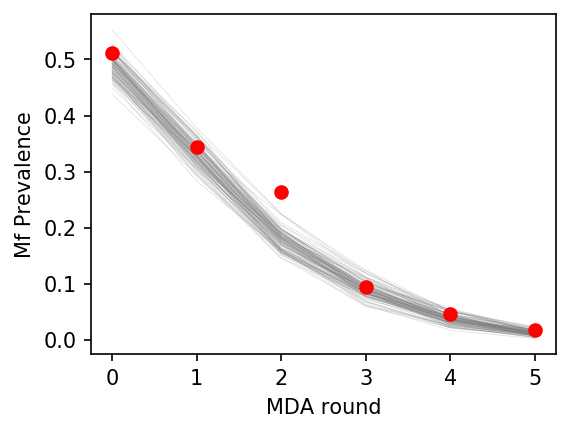
**

**Figure S4: 100 realisations of TRANSFIL, with MAP parameters (*k* = 0.48, *V:H = 101.0*, *a_max_* = 20.3 , *r = 0.24*) and Ngahmbule baseline data (red).**

**
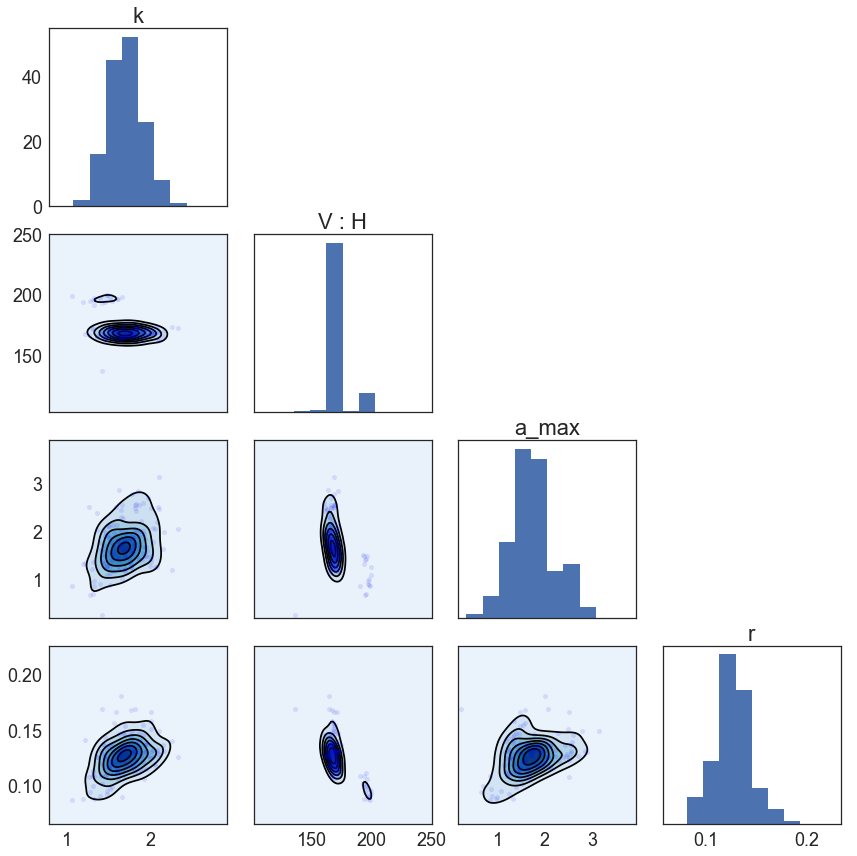
**

**Figure S5: ABC parameter fits for Yauatong (PNG) dataset.**

**
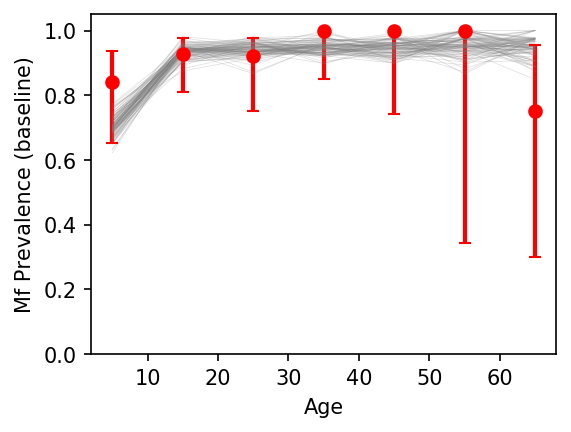

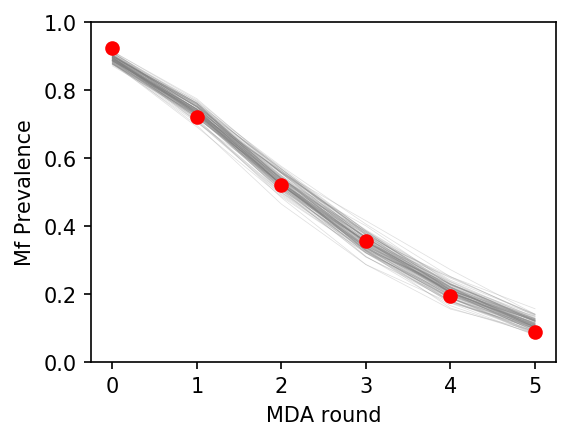
**

**Figure S4: 100 realisations of TRANSFIL, with MAP parameters (*k* = 1.66, *V:H = 167.1*, *a_max_* = 1.7, *r = 0.12*) and Yauatong baseline data (red).**

References

1. Singh BK, Bockarie MJ, Gambhir M, Siba PM, Tisch DJ, Kazura J. Sequential modelling of the effects of mass drug treatments on anopheline-mediated lymphatic filariasis infection in papua new guinea. PLoS One. 2013;8. doi: 10.1371/journal.pone.0067004.

2. Smith ME, Singh BK, Irvine MA, Stolk WA, Subramanian S, Hollingsworth TD, et al. Predicting lymphatic filariasis transmission and elimination dynamics using a multi-model ensemble framework. Epidemics. 2017;18:16-28. doi: <https://doi.org/10.1016/j.epidem.2017.02.006>.
